# Interpreting Modulation Transfer Function in Endoscopic Imaging: Spatial-Frequency Conversion Across Imaging Spaces and the Digital Image Domain with Case Studies

**DOI:** 10.3390/s26030827

**Published:** 2026-01-27

**Authors:** Quanzeng Wang

**Affiliations:** Center for Devices and Radiological Health, U.S. Food and Drug Administration, Silver Spring, MD 20993, USA; quanzeng.wang@fda.hhs.gov

**Keywords:** video endoscope, spatial resolution, image dimensions, pixel resolution, modulation transfer function, MTF, spatial frequency response (SFR), spatial frequency, angular spatial frequency, ISO 8600-5, slanted edge, ISO 12233, local magnification, picture height, pixel pitch, scaling factor, resample, geometric distortion, object plane, image sensor plane, digital image domain, object space, image space, imaging chain

## Abstract

Endoscopes are widely used in medicine, making objective evaluation of imaging performance essential for device development and quality assurance. Image resolution is commonly characterized by the modulation transfer function (MTF); however, its interpretation depends critically on how spatial frequency is defined and reported. Because spatial frequency is directly tied to sampling, it can be expressed in different units across the imaging chain, including the object plane, image sensor plane, and digital image domain. Inconsistent conversion between these spaces and domains can mislead comparisons and even alter the apparent ranking of regions of interest (ROIs) or imaging systems. This work presents a systematic analysis of spatial-frequency relationships along the endoscopic imaging chain and provides a practical conversion and interpretation workflow for MTF analysis. The framework accounts for sensor sampling, in-camera processing, resampling or scaling, and geometric distortion. Because geometric distortion introduces position-dependent sampling across the field of view, ROI-specific local-magnification measurements are incorporated to convert measured MTFs to a consistent object space spatial-frequency axis. Two case studies illustrate the implications. First, an off-axis ROI may appear to outperform the image center when MTF is expressed in digital image domain cycles per pixel, but this conclusion reverses after conversion to object space cycles per millimeter using local magnification. Second, resampled image outputs can yield inflated MTF curves unless scaling differences between formats are explicitly incorporated into the spatial-frequency axis. Overall, the proposed conversion and reporting workflow enables consistent and physically meaningful MTF comparison across devices, ROIs, and acquisition configurations when geometric distortion, sampling, or resampling differs, clarifying how optics, sensor characteristics, and image processing jointly determine reported MTF results.

## 1. Introduction

Endoscopes play a crucial role in clinics and hospitals, aiding in the early detection of cancer and the diagnosis of diseases, thereby improving patient care. Millions of endoscopic procedures performed monthly continue to propel advancements in endoscopy. The imaging performance of endoscopes, which can be measured using metrics like resolution [1], geometric distortion [2], field of view [3], uniformity of image intensity [4], and noise [5], is fundamental to their effective utilization. Resolution, in particular, holds significant importance as it determines the ability of an endoscope to depict anatomical details, including small structures related to abnormal conditions. Therefore, accurately assessing resolution is vital for ensuring the reliability of endoscopic procedures. Recent advances in endoscopy also illustrate the growing need for reliable, quantitative characterization of imaging performance. For example, MHz-OCT-based real-time 3D endoscopy has been demonstrated for rectal disease screening [6], highlighting the importance of accurate spatial-resolution assessment in emerging 3D and live-imaging modalities.

The modulation transfer function (MTF) serves as a comprehensive metric for evaluating spatial resolution of images captured by a digital imaging system, including endoscopes [1,7]. It measures modulation loss as a function of spatial frequency in digital images. The term “spatial frequency response (SFR)” is often used in place of MTF because digital cameras are generally not strictly linear systems. However, consistent with common usage in endoscopic imaging, the measured SFR is referred to as the MTF throughout this article. MTF has become a standard metric for characterizing the optical performance of imaging systems, and several international standards on MTF measurement have been developed [1,8,9,10,11,12,13,14,15]. Among these standards, the ISO 8600-5 standard [1] is the only one specifically dedicated to endoscope MTF measurement. This standard applies to rigid medical endoscopes with optics and specifies methods for measuring their optical resolution. However, it excludes the most widely used opto-electronic video endoscopes [16] and does not fully address their unique characteristics. In particular, video endoscopes introduce several complexities not covered by ISO 8600-5: (1) sampling is determined by the discrete sensor-pixel grid rather than the optical image alone; (2) in-camera image-processing steps—such as gamma correction, edge enhancement, noise reduction, and automatic gain control—modify the native sensor signal; and (3) rescaling or resampling during image formatting and geometric distortion correction change the relationship between image sensor plane and digital image domain spatial frequencies. These characteristics make it necessary to analyze spatial-frequency definitions and MTF interpretation across the imaging chain in a consistent framework.

Recently, we investigated methods to measure the MTF of video endoscopes [16]. In that study, all MTF curves are expressed as a function of spatial frequency in cycles per millimeter in the object plane. That work focused on establishing methods for video-endoscope MTF measurement but did not address an important remaining limitation: object plane frequencies alone do not explain how sensor sampling limits, in-camera scaling or resampling, and spatially varying magnification caused by geometric distortion influence the interpretation of MTF curves. These factors determine how spatial frequencies in different imaging spaces or domains relate to one another and can lead to contradictory conclusions if not treated consistently.

Spatial frequency can be expressed in different units and refer to different imaging spaces or domains (typically object plane, image sensor plane, and digital image domain). In the imaging chain, the object plane is where the scene or object resides, providing the spatial details that need to be captured. Light emitted or reflected from an object passes through the optical system and is then focused onto the image plane to form an image. The image plane is a theoretical surface where the image would be perfectly formed. The image sensor plane is the actual physical surface where the image is captured by the sensor and ideally coincides with the image plane. Finally, the image from the image sensor plane is converted into a digital format in the digital image domain, where it is processed, analyzed, and stored. Thus, the object plane provides the original spatial information, the image plane is where the ideal image focus occurs, the image sensor plane captures this focused image, and the digital image domain is where the image data is digitally processed and utilized. For MTF analysis, the object plane, image sensor plane, and digital image domain are critical in evaluating system performance. MTF curves based on spatial frequencies with different units and in different spaces or domains of the imaging chain can differ, which is important to know when comparing two MTF curves. In practice, inconsistent or incorrect use of spatial-frequency units can lead to misinterpretation of MTF results. For example, during colonoscope procurement or routine quality-assurance review, a device may appear to meet resolution requirements when frequencies are expressed in cycles per image pixel but fall short when evaluated in object space units that correspond to clinically relevant textures such as subtle mucosal structures. Such discrepancies can affect acceptance or rejection decisions and underscore the need for a consistent spatial-frequency framework.

While there is limited information on converting between different spatial frequencies, to our knowledge, no prior work has provided a systematic, integrated discussion on these spatial frequencies and their effects on the interpretation of MTF results. This paper extends the test methods in Ref. [16] by presenting a unified conversion framework linking spatial-frequency definitions across the object plane, image sensor plane, and digital image domain, and by incorporating local magnification to account for spatially varying geometric distortion in endoscopic systems. We also briefly discuss the commonly used angular spatial frequency in object space. Additionally, we present two case studies that demonstrate how spatial-frequency definitions and parameter choices can alter MTF interpretation. These results highlight the importance of selecting appropriate spatial-frequency units and parameters, and of correctly identifying the imaging space or domain when reporting MTF results.

## 2. Conversion of Spatial Frequencies Across Imaging Spaces and Domains

### 2.1. Spatial Frequencies

The resolution of digital imaging systems can be characterized in two ways: image dimensions (or pixel resolution) and spatial resolution. Image dimensions describe an image’s width and height in pixels, whereas spatial resolution describes an imaging system’s ability to resolve spatial information (as quantified by spatial frequency) in the object or scene being imaged. The spatial resolution of an optical imaging system (like a camera lens, microscope, or rigid endoscope) without digital components is referred to as optical resolution. In digital imaging systems, while a higher pixel resolution may contribute to improved spatial resolution, spatial resolution is also limited by the quality of the optics and the sensor. Therefore, spatial resolution, rather than image dimensions, should be used to evaluate the resolution of digital imaging systems.

MTF is widely used to quantify the spatial resolution of a digital imaging system. An MTF plot shows the modulation transfer factor (a measure of how well modulation is transferred from the object to the image [1]) as a function of spatial frequency, a metric that typically quantifies how many cycles (cy) are present per unit of distance (or angle for angular spatial frequencies). Spatial frequency has different values and units across the imaging chain. The remainder of this subsection establishes the practical spatial-frequency conversion framework—across the digital image domain, image sensor plane, and object plane—that will be applied in later sections and case studies.

Spatial frequencies in the image sensor plane and digital image domain can be measured in cycles per sensor pixel (cy/pix,sen) or per image pixel (cy/pix,im), depending on whether referring to the physical sensor or the final digital representation. The image sensor pixel (also known as a photo-site or sensor element) is the fundamental physical component on an imaging sensor, such as a CCD or CMOS. It captures light and converts it into an electrical signal, generating raw analog data that are later digitized (quantized and stored) to form a digital image. The physical size of a sensor pixel, known as the pixel pitch ($P_{mm/pix,sen}$ or $P_{\mu m/pix,sen}$), is typically measured in millimeters (mm) or micrometers (µm). In contrast, a digital image pixel is the smallest unit of a digital image. The unit cycles per pixel (cy/pix) are often used for both sensor pixel and image pixel. To avoid confusion, we use the symbols of cy/pix,sen and cy/pix,im to distinguish between cycles per sensor pixel and cycles per image pixel, respectively. Therefore, the spatial frequencies in the image sensor plane and digital image domain can be expressed as $f_{cy/pix,sen}$ and $f_{cy/pix,im}$.

The total number of sensor pixels and image pixels may differ if resampling occurs during image processing. The degree of resampling can be quantified by a scaling factor (or resampling rate), s, defined as the ratio of the output image dimension in pixels ($N_{pix,im}$) to the image sensor’s native pixel dimensions ($N_{pix,sen}$):

(1) $$s=N_{pix,im}/N_{pix,sen}$$

where $N_{pix,im}$ represents the number of pixels along a given dimension in the output image, and $N_{pix,sen}$ represents the number of active pixels along the same dimension in the sensor’s native pixel array. In some cases, the image may be cropped, resulting in fewer active sensor pixels than the total available sensor pixels. s>1 indicates upsampling and s<1 indicates downsampling.

The resampling can be isotropic, where s values along the x and y directions ($s_{x}$ and $s_{y}$) are the same, or anisotropic, where $s_{x}$ and $s_{y}$ are different. For example, if the image sensor has 600 × 400 pixels (width × height) and the output image has 300 × 1200 pixels, the scaling factors are $s_{x}$ = 0.5 (300/600, downscaling in width) and $s_{y}$ = 3 (1200/400, upscaling in height). In more complex scenarios, $s_{x}$ and/or $s_{y}$ can vary across different regions of the image. For instance, in an endoscope image with significant barrel distortion, applying distortion correction can result in spatially varying s that changes with the radial distance from the image center [2]. Equation (1) defines a single global s (or $s_{x}$ and $s_{y}$ for anisotropic resampling) for simplicity. However, after distortion correction or other spatially varying resampling operations, s may change across the image. In such cases, a single global s cannot fully represent local sampling behavior. Therefore, when spatially varying scaling is present, local s should be used for MTF analysis whenever possible. If only a global s is available, the resulting spatial-frequency conversion should be interpreted with caution, as it may not fully capture position-dependent sampling changes.

Since MTF is calculated from digital images using image pixels as the fundamental unit, spatial frequency for MTF curves is often expressed in the unit of cy/pix,im ($f_{cy/pix,im}$). The relationship between $f_{cy/pix,im}$ and $f_{cy/pix,sen}$ can be expressed as:

(2) $$f_{cy/pix,sen}=s\cdot f_{cy/pix,im}$$

where s may vary with direction or location if the scaling is anisotropic.

The Nyquist frequency ($f_{Nyq}$) is defined as the highest frequency that can be accurately sampled without aliasing, and it depends on the sampling grid (sensor pixel or image pixel) that limits spatial resolution. In practical terms, two scenarios arise. If the final image is upsampled from the raw sensor image (i.e., s>1), $f_{Nyq}$ is 0.5 cy/pix,sen or (0.5/s) cy/pix,im. In this case, interpolation adds additional image pixels, but no new spatial information is introduced; thus, resolution remains limited by the sensor. Conversely, if the final image is downsampled from the raw sensor image, $f_{Nyq}$ is 0.5 cy/pix,im or (0.5·s) cy/pix,sen. Here, the image is spatially compressed by combining or discarding sensor data, and the spatial resolution is limited by the final image grid. These distinctions indicate which $f_{Nyq}$ value should be used when interpreting MTF curves under typical endoscopic imaging conditions. Accordingly, an MTF plot only needs to present values at spatial frequencies up to $f_{Nyq}$.

If $P_{mm/pix,sen}$ is known, $f_{cy/pix,sen}$ can be converted to frequency in the unit of cycles per mm in the image sensor plane as

(3) $$f_{cy/mm,sen}=f_{cy/pix,sen}/P_{mm/pix,sen}$$

Spatial frequency is sometimes expressed in the unit of cycles per picture height ($f_{cy/H}$). Picture height typically refers to the vertical size of an image or display, usually measured in pixels for image sensor ($H_{pix,sen}$) or digital image ($H_{pix,im}$) or in physical units like mm for image sensor ($H_{mm,sen}$) or printed/displayed images ($H_{mm,im}$). $f_{cy/H}$ can be calculated with either of the following equations:

(4) $$f_{cy/H}=f_{cy/pix,sen}\cdot H_{pix,sen}$$

(5) $$f_{cy/H}=f_{cy/pix,im}\cdot H_{pix,im}$$

(6) $$f_{cy/H}=f_{cy/mm,sen}\cdot H_{mm,sen}$$

Spatial frequency can also be expressed in the unit of linewidths per picture height ($f_{line/H}$). Since one cycle is equal to two linewidths, $f_{line/H}=2f_{cy/H}$. Both $f_{cy/H}$ and $f_{line/H}$ are included here for conceptual background and completeness, not for direct use in the quantitative analyses that follow, as they are widely used in imaging standards such as ISO 12233:2024 [8]. Therefore, these units are not used in the Section 4 case studies, which focus on more commonly applied $f_{cy/pix,im}$ and $f_{cy/mm,ob}$. Using the conversion equations provided in this paper, readers can convert spatial frequencies between units and across imaging spaces and domains as needed.

In addition to the digital image domain, spatial frequency can be defined in both the image sensor and object planes. While both are useful in different contexts, object plane spatial frequency is often more informative for assessing imaging system performance. First, it has a direct physical interpretation, corresponding to real object features (e.g., anatomical structures), which facilitates evaluation of the system’s ability to resolve fine details. Second, it is independent of system-specific factors such as optical magnification and image sampling, enabling more consistent comparison and analysis across imaging systems or settings. Therefore, accurate conversion of spatial frequency from the image sensor plane to the object plane is essential.

The main parameter for converting spatial frequency from the image sensor plane to the object plane is the lateral (or transverse) magnification (M), which is defined as the ratio of an object’s image size (height or width) as seen through an optical system (like a microscope, telescope, or camera) to the object’s actual size, measured perpendicular to the optical axis. It describes how much larger or smaller the object appears through an optical system. The longitudinal (or axial) magnification—defined as the ratio of image length to object length when the object is aligned with the optical axis—is not addressed in this paper. Unless otherwise specified, magnification refers to lateral magnification in this paper. The definition of M has been extended to digital imaging, where it represents the ratio of an object’s image size on the camera sensor to its actual size in the object plane. Another term called image scale (*IS*) refers to the ratio between the size of an object in an image and the object’s actual size. If the images are on the image sensor, M and *IS* are the same. However, *IS* can also be used for scenarios of images on a display or in a printout. For the conversion of spatial frequencies between the image sensor plane and object plane, we adopt the definition of M in digital imaging.

Endoscopes usually exhibit significant geometric distortion, resulting in varying M across the object or sensor plane. The M within an infinitesimally small region is referred to as the local magnification ($M_{L}$). $M_{L}$ depends on target distance and may vary across the object or image sensor plane in systems with geometric distortion. It should therefore be measured at the same working distance and within the same ROI used for MTF measurement to ensure accurate conversion of spatial frequency from the image sensor plane to the object plane. The object plane spatial frequency in cycles per mm, $f_{cy/mm,ob}$, can be converted from $f_{cy/mm,sen}$ as

(7) $$f_{cy/mm,ob}=f_{cy/mm,sen}\cdot M_{L}$$

Endoscope MTF is often measured using a slanted-edge target [1,8,16], whereas other imaging modalities may employ different targets (e.g., a dead-leaves target for mobile phone MTF [17]). The default spatial frequency derived from the target image is $f_{cy/pix,im}$, which should first be converted to $f_{cy/pix,sen}$ (Equation (2)), and then to $f_{cy/mm,ob}$ using the following equation,

(8) $$f_{cy/mm,ob}=\;f_{\;cy/pix,sen}\cdot \frac{M_{L}}{P_{mm/pix,sen}}$$

While $P_{mm/pix,sen}$ is often unknown to users, the value of $M_{L}/P_{mm/pix,sen}$, can be measured by capturing images of a short segment of a target (e.g., a ruler with high resolution and accuracy) and calculated as

(9) $$\frac{M_{L}}{P_{mm/pix,sen}}=\frac{N_{pix,sen}}{L}=\frac{N_{pix,im}}{s\cdot L}$$

where L is the length of the short target segment (in mm), $N_{pix,sen}$ and $N_{pix,im}$ are the numbers of pixels corresponding to this length on the image sensor and in the digital image, respectively, and s is the scaling factor. If the target segment is positioned vertically from the top edge to the bottom edge, $N_{pix,sen}{=H}_{pix,sen}$ and $N_{pix,im}=H_{pix,im}$.

The value of $M_{L}/P_{mm/pix,sen}$ depends on target distance, but a detailed discussion is beyond the scope of this paper. For a typical endoscope with significant geometric distortion, $M_{L}$ also varies across the object plane. Thus, a value of $M_{L}/P_{mm/pix,sen}$ measured with this method reflects an average $M_{L}$ over the target segment. For conversion between $f_{\;cy/pix,sen}$ and $f_{cy/mm,ob}$ at specific locations, $M_{L}/P_{mm/pix,sen}$ should be measured at each location. Alternatively, one may measure $M_{L}/P_{mm/pix,sen}$ at the image center and derive values at other locations using a normalized $M_{L}$ curve, as discussed in a journal paper [2] and Section 3.

$M_{L}/P_{mm/pix,sen}$ has units of sensor pixels per object mm (pix,sen/mm,ob), indicating how many sensor pixels correspond to 1 mm of linear distance on the object. As an example, we measured $M_{L}/P_{mm/pix,sen}$ of our endoscope at ROIs A and B_2_ (Section 3.1) at 80 mm target distance. The results are 8.93 pix,sen/mm,ob at A and 7.39 pix,sen/mm,ob at B_2_ when s is 1. Therefore, $f_{Nyq}$ of 0.5 cy/pix,sen in the image sensor plane corresponds to 4.47 cy/mm,ob and 3.70 cy/mm,ob in the object plane for ROIs A and B_2_, respectively, based on Equation (8).

### 2.2. Angular Spatial Frequencies

Angular spatial frequency represents how many intensity cycles occur per unit viewing angle. In endoscopic imaging, it quantifies spatial detail with respect to the system’s angular coverage rather than linear distance. Because multiple standards reference angular spatial frequency without providing equations linking it to other spatial-frequency definitions, it is included here primarily for conceptual completeness.

Angular spatial frequency is often used within the object space field of view (FOV) in units of cycles per radian ($f_{cy/rad,ob}$) or cycles per degree ($f_{cy/deg,ob}$). The conversion from spatial frequency to angular spatial frequency is based on geometric and trigonometric principles, specifically through the differentiation of an inverse trigonometric function:

(10) $$d(\tan^{-1}x)=\frac{1}{1+x^{2}}dx\;$$

where $-\pi /2<\tan^{-1}x<\pi /2$.

**Figure 1:**
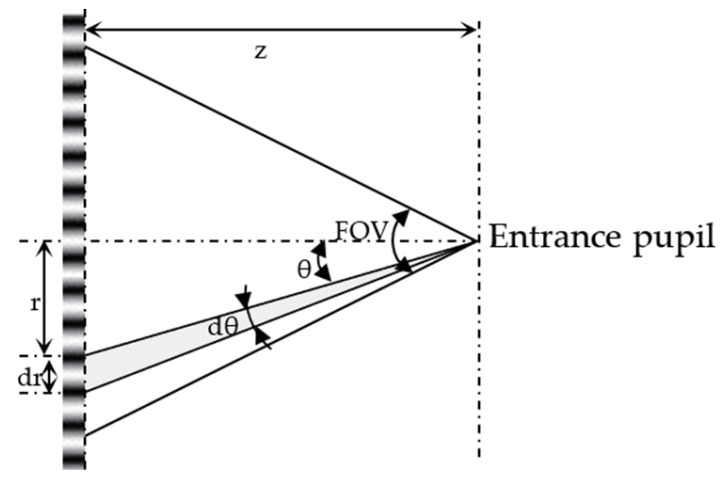
Geometry for conversion between spatial frequency and angular spatial frequency in object space.

As illustrated in Figure 1, the distance between the entrance pupil and the target is z. The viewing angle θ at radius r on the object plane is $\theta =\tan^{-1}\left(r/z\right)$. The differential of θ is

(11) $$d\theta =d(\tan^{-1}\frac{r}{z})=\frac{1}{1+{\left(\frac{r}{z}\right)}^{2}}\cdot d(\frac{r}{z})=\frac{z}{z^{2}+r^{2}}dr$$

$f_{cy/mm,ob}$ at radius r can be understood as the number of cycles within dr (*N*) divided by dr, i.e., $f_{cy/mm,ob}=N/dr$. The angular spatial frequency in the unit of cy/rad,ob at the same location can be calculated as $f_{cy/rad,ob}=N/d\theta$. Therefore,

(12) $$f_{cy/rad,ob}=\frac{N}{d\theta }=\frac{N}{\frac{z}{z^{2}+r^{2}}dr}=\frac{z^{2}+r^{2}}{z}\cdot \frac{N}{dr}=(z+\frac{r^{2}}{z})\cdot f_{cy/mm,ob}$$

When r is small (e.g., close to the FOV optical axis), the above equation can be simplified as $f_{cy/rad,ob}=z\cdot f_{cy/mm,ob}$. The $f_{cy/deg,ob}$ can be calculated as $f_{cy/deg,ob}=(\pi /180)\cdot f_{cy/rad,ob}$. While this section mainly discusses the relation between spatial frequencies and angular spatial frequency in object space. The equations can also be extended to image space.

Converting spatial frequency to angular spatial frequency requires knowledge of the distance z between the entrance pupil and the target (Figure 1). In practical endoscopic systems, the entrance-pupil location is often unknown to users but can be measured in principle. A detailed description of entrance-pupil measurement is beyond the scope of this paper; readers are referred to our previous FOV study [3], where Equation (7) and Figure 5 illustrate a practical measurement approach. Measurement errors may arise from manufacturing tolerances or alignment issues during testing. As a result, uncertainty in z can propagate into the calculation of angular spatial frequency, and care should be taken when applying these conversions in practice.

### 2.3. Summary of Conversion Equations

**Figure 2:**
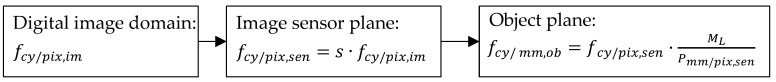
Flowchart of essential spatial-frequency conversion equations.

We have discussed the conversions of spatial frequency between different units and across the digital image domain, image sensor plane and object plane, as well as the derivation of angular spatial frequency from spatial frequency. Understanding these principles and equations is important for MTF interpretation. Table 1 summarizes the relationships among spatial and angular spatial frequencies and the associated parameters. In practical MTF analysis, a slanted-edge MTF measured in the digital image domain is typically converted through the sequence $f_{cy/pix,im}$ → $f_{cy/pix,sen}$ → $f_{cy/mm,ob}$, where the final step uses the ${M_{L}/P}_{mm/pix,sen}$ term to obtain spatial frequencies in the object plane. Figure 2 illustrates the key conversion equations linking the digital image domain, image sensor plane and object plane.

Several conversion equations require the value of $P_{mm/pix,sen}$, which is often unknown to users. In such case, $M_{L}/P_{mm/pix,sen}$ can be measured by imaging a short target segment with known length at a defined ROI and measuring distance. The value of $M_{L}/P_{mm/pix,sen}$ is a function of target distance and lateral location. If the captured images have pixel dimensions different from the image sensor’s pixel dimensions due to resampling, $f_{Nyq}$ is 0.5 cy/pix,im or (0.5·*s*) cy/pix,sen for downsampling and 0.5 cy/pix,sen or (0.5/s) cy/pix,im for upsampling.

## 3. Measurement of Endoscope Local Magnification ($M_{L}$)

As discussed in Section 2, $M_{L}$ is a key parameter for converting spatial frequencies between the image sensor plane and the object plane (Equations (7)–(9)). Endoscopes usually exhibit significant geometric distortion, resulting in varying $M_{L}$ across different object distance, lateral positions, and directions (e.g., radial, and tangential) [2]. Because accurate MTF interpretation requires correct mapping of spatial frequencies between the image sensor and object planes, the variation in $M_{L}$ must be measured and incorporated. The need to account for this spatially varying $M_{L}$ motivates the case studies but does not restrict the applicability of the framework to endoscopes alone.

The $M_{L}$ method [2] generates a curve showing normalized $M_{L}$ as a function of radius through a fitted equation derived from all measured data points and then normalized based on the value at the center. The absolute $M_{L}$ at any radius can be obtained by multiplying the normalized $M_{L}$ curve by the measured $M_{L}$ at center ($M_{C}$). These absolute $M_{L}$ values can then be used to convert spatial frequencies across different imaging spaces or domains according to Equations (7)–(9). The following subsections describe how the normalized $M_{L}$ curve is generated and how $M_{C}$ is measured.

### 3.1. Measurement of Normalized $M_{L}$

In a previous study, we developed a method to measure $M_{L}$ as a function of radial position perpendicular to the optical axis [2]. The $M_{L}$ along a radial direction from the FOV center is called local radial magnification ($M_{LR}$), and the $M_{L}$ along the direction tangentially oriented to a radial direction is called local tangential magnification ($M_{LT}$). At the center of the FOV, $M_{LR}$ and $M_{LT}$ are the same, called center magnification $M_{C}$. At other locations, $M_{LR}$ and $M_{LT}$ might be different and can be expressed as polynomial functions with radius as the variable. Since endoscopic imaging is not based on perspective projection that renders a straight line in object space as a straight line in the image, the $M_{LR}$ and $M_{LT}$ equations based on the radius in the object plane (undistorted radius, $R_{u}$) and in the digital image domain (distorted radius, $R_{d}$) are different. The digital image domain and the image sensor plane contain equivalent information regarding geometric distortion when the captured images have not undergone non-linear distortion correction or geometric warping; this equivalence may not hold once such processing is applied.

**Figure 3:**
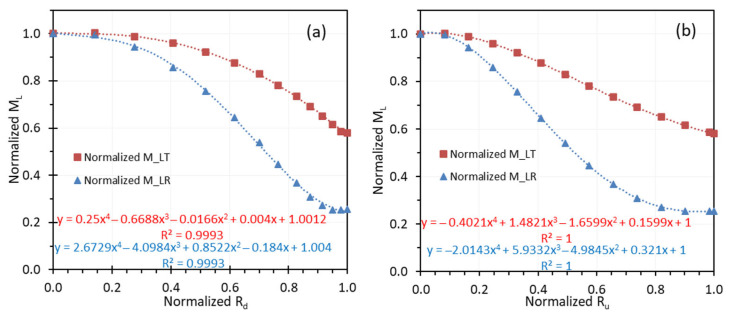
Normalized *M_LT_* and *M_LR_* as a function of (**a**) normalized *R_d_* in the digital image domain and (**b**) normalized *R_u_* in the object plane.

Figure 3 illustrates the polynomial fitting curves and equations of normalized $M_{LR}$ and $M_{LT}$ as functions of normalized $R_{u}$ or $R_{d}$ for our endoscopic system (EVIS EXERA II, Olympus America, Center Valley, PA, USA) that includes a high-intensity xenon light source (CLV-180), a gastrointestinal videoscope (GIF-H180), and a video system center (CV-180). For normalized $R_{d}$, the distance from the center to the left or right edge of the chart images is normalized to 1. The distance in the object plane corresponding to the normalized $R_{d}$ value of 1 is considered as normalized $R_{u}$ value of 1. The figure highlights that the curve shapes differ depending on whether the normalized radius is in the digital image domain (Figure 3a) or the object plane (Figure 3b). Methods for obtaining these curves have been provided in our previous paper [2]. In that paper, a fifth-degree polynomial was used to fit the distortion curve, achieving R^2^ values of at least 0.999. This approach has also been applied to several rigid endoscopes (data not published) and has demonstrated robust performance. The required polynomial degree or functional form of fitting equations, however, may vary depending on the R^2^ threshold selected for a given application.

**Figure 4:**
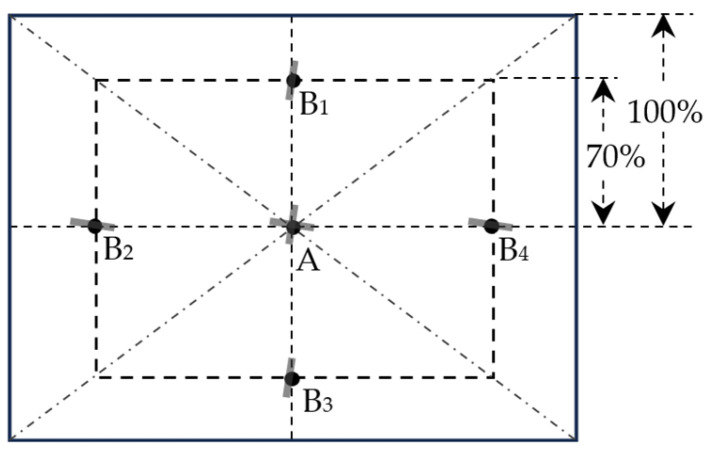
ROIs on chart images for endoscope MTF calculation. The on-axis point (A) is at the image center, and off-axis points (B_1_–B_4_) are at 70% of the distances from A to the image edges [16]. Short, thick gray bars mark the target edge direction at these points.

It is more convenient to calculate $M_{LR}$ and $M_{LT}$ at digital image domain using the polynomial equations in Figure 3a. $M_{LR}$ or $M_{LT}$ can then be used to convert spatial frequencies in the image sensor plane and in the object plane. However, it is essential to understand which equation should be used. Endoscope MTF should be measured at five image locations, as illustrated in Figure 4. The edge chart is aligned such that the edge in the image lies approximately along radial direction from the optical axis, ensuring that the edge is straight. Since spatial frequency is perpendicular to the edge, the MTF curves are along tangential direction (approximately horizontal at $B_{1}$ and $B_{3}$, approximately vertical at $B_{2}$ and $B_{4}$, and either orientation at A, each with a small tilt). Therefore, the $M_{LT}$ equation in red in Figure 3a should be used. In this equation, y is normalized $M_{LT}$ and x is normalized $R_{d}$ with the distance from center to left or right edge as 1. At position A, the normalized $R_{d}$ is 0 and the normalized $M_{LT}$ is 1. At position $B_{2}$, the normalized $R_{d}$ is 0.7, and the normalized $M_{LT}$ is 0.83. In our previous study, we have shown that the curves of absolute $M_{LT}$ and normalized $M_{LT}$ as a function of normalized $R_{d}$ can overlap by adjusting the scales of their y coordinates (Figure 10 in [2]). Therefore, if we know the actual $M_{LT}$ at center A (i.e., $M_{C}$), the actual $M_{LT}$ at $B_{2}$ will be 0.83 times $M_{C}$.

It should be noted that the polynomial coefficients shown in Figure 3 were derived specifically for the endoscopic system used in this study. These coefficients are not expected to generalize to other endoscope models or even to different units of the same model, as $M_{L}$ behavior depends on the detailed optical design and manufacturing tolerances. The fitting procedure itself is general, but each imaging system should have its own $M_{L}$ curves measured and fitted to obtain accurate conversion parameters for spatial-frequency analysis.

### 3.2. Measurement of $M_{C}$

In general, the magnification of a lens is influenced by both its focal length (f) and the subject distance to the entrance pupil (z). Under the paraxial approximation, their relationship is given by: M=f/(z−f). In general, a closer distance results in higher M, but M is not directly inversely proportional to z, except for the scenario of z≫f. While a simple prime lens has a fixed f, the f of a zoom lens can vary.

Since an endoscope exhibits significant geometric distortion, it cannot be simplified as a single lens, and its f is generally unknown. Therefore, the users often need to measure $M_{L}$ (including $M_{LR}$ and $M_{LT}$) directly. As discussed earlier, once $M_{C}$ is measured and the normalized $M_{LR}$ and $M_{LT}$ equations are known, absolute $M_{LR}$ and $M_{LT}$ at any radial position can be determined by multiplying $M_{C}$ by the corresponding normalized $M_{LR}$ and $M_{LT}$.

As discussed in Section 2.1, $M_{L}$ is defined as the ratio of the size of the image on the image sensor to the actual size of a small object segment. To calculate $M_{L}$, one needs to know the length of the segment, the pixel number of the segment image, scaling factor (or the pixel number of the segment on the image sensor), and the pixel pitch of the sensor ($P_{mm/pix,sen}$). However, $P_{mm/pix,sen}$ is sometimes unavailable. In such cases, the $M_{L}$-to-$P_{mm/pix,sen}$ ratio ($M_{L}/P_{mm/pix,sen}$) can be treated as a single measurable quantity, as shown in Equation (9).

$M_{C}$ and $M_{C}/P_{mm/pix,sen}$ are $M_{L}$ and $M_{L}/P_{mm/pix,sen}$ at the image center. In practice, they are often estimated by measuring a short target segment within a small center region that is assumed to be free of distortion [18]. However, there is no consensus on the appropriate size of this distortion-free center region, and the optimal choice likely depends on the distortion characteristics of the imaging system. Using too short a target segment increases susceptibility to reading and pixel-sampling error, whereas using a longer segment may violate the assumption of negligible distortion at the center. As a practical guideline, the assumed undistorted center region should be within 10% of the image width (or height, whichever is longer). To reduce measurement error, sharp images of short target segments (grid target, dot target, ruler, etc.) at the center should be used and multiple measurements can be averaged.

We measured $M_{C}$ or $M_{C}/P_{mm/pix,sen}$ by imaging grid targets with known grid sizes. The targets were aligned perpendicular to the endoscope optical axis, and the distance between the endoscope distal end and the target was adjustable. Targets with different grid sizes (e.g., 0.5 mm × 0.5 mm, 1.0 mm × 1.0 mm) could be used at various distances, with smaller grids preferred at shorter distances where $M_{C}$ is higher. Only the center region of the target needed to be imaged. The captured images were then analyzed to calculate $M_{C}$ or $M_{C}/P_{mm/pix,sen}$.

**Figure 5:**
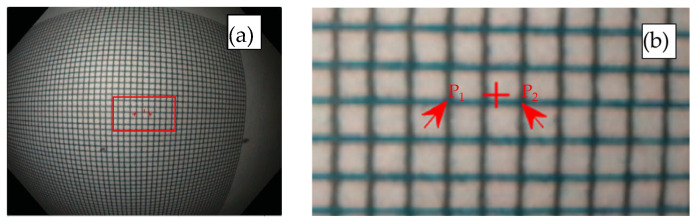
Images of a grid chart with a grid size of 0.5 mm × 0.5 mm at a distance of 14 mm. (**a**): the whole image; (**b**): the center region. The two red arrows point to the two corners near the center, P_1_ and P_2_, which were used to calculate *M_C_*.

Figure 5 shows the image of a grid target with a grid size of 0.5 mm × 0.5 mm at a distance of 14 mm. The scaling factor is 1. The red cross in the figure indicates the image center. To calculate $M_{C}$, two corners near the center ($P_{1}$ and $P_{2}$) were identified as indicated by the two red arrows; and their pixel coordinates were read (using MATLAB or other software). The distance between $P_{1}$ and $P_{2}$ was then calculated in pixels, which in this case is 56 pixels. Assuming a pixel pitch of $P_{mm/pix,sen}$ = 2.8 microns, the distance between $P_{1}$ and $P_{2}$ in the image sensor plane was calculated as 0.157 mm (0.0028 × 56). Since the distance between these two points is 1 mm on the grid target, $M_{C}$ was calculated as 0.157. $M_{C}/P_{mm/pix,sen}$ can be calculated as 56 pix,sen/mm,ob based on Equation (9). Repeating the measurement and averaging the results can reduce error. The same process can be used to measure $M_{C}$ or $M_{C}/P_{mm/pix,sen}$ at different target distances. Analogous to the absolute $M_{LR}$ and $M_{LT}$, the absolute $M_{LR}/P_{mm/pix,sen}$ and $M_{LT}/P_{mm/pix,sen}$ at any radial position can be obtained by multiplying $M_{C}/P_{mm/pix,sen}$ by the corresponding normalized $M_{LR}$ and $M_{LT}$.

**Figure 6:**
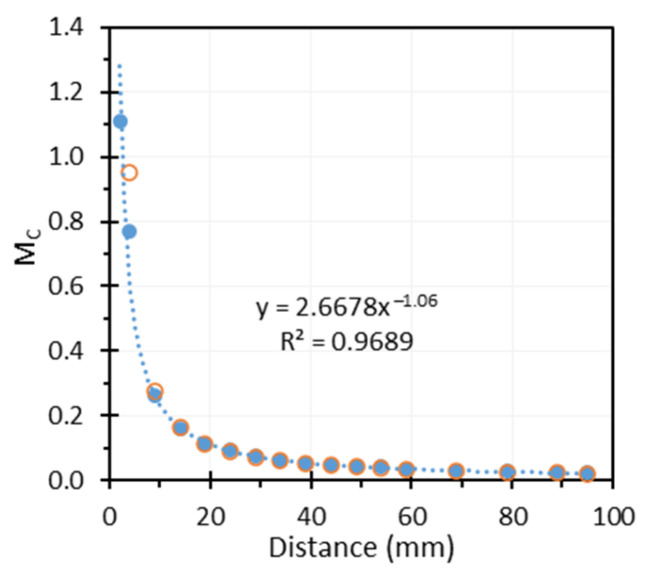
*M_C_* as a function of distance (blue dots: measured *M_C_*; blue line: trendline of the measured *M_C_*; orange circles: calculated *M_C_* based on the equation of M=f/(z−f), assuming f = 1.95 mm).

The measured $M_{C}$ values for our endoscope at different target distances are shown as the blue dots in Figure 6. The blue dashed line represents the trendline based on a power function. From the trendline equation, the $M_{C}$ at 80 mm is calculated as 0.026. This value, when multiplied by the normalized $M_{LT}$ of 0.83 calculated in Section 3.1, gives 0.021, which is the absolute $M_{LT}$ at $B_{2}$. Therefore, the absolute $M_{LT}$ values of 0.026 and 0.021 should be used for A and $B_{2}$, respectively, in Equations (7)–(9)*,* when calculating spatial frequency in the object plane.

The measured $M_{C}$ curve can also be used to estimate the endoscope’s focal length. The orange circles in Figure 6 represent M values calculated based on the lens function of M=f/(z−f), assuming a focal length of 1.95 mm. Since the orange circles and blue dots nearly overlap, the endoscope’s focal length can be estimated to be approximately 1.95 mm, assuming a pixel pitch of 2.8 microns and no resampling during image processing (i.e., scaling factor of 1).

## 4. Two Case Studies

We performed two case studies to demonstrate how MTF results can vary when different spatial-frequency units or parameters are used in the calculation. The same endoscopic system described in Section 3 was used to capture digital images of slanted-edge targets for MTF evaluation. All operating parameters were set according to the recommendations in Table 4 of our previous paper [16]. For each ROI, images were captured under identical conditions, including sufficient uniformity of image luminance, high-quality test chart (ISO 12233:2017 [19] edge-SFR chart, Imatest LLC, Boulder, CO, USA), linearized image data, disabled auto-gain control, disabled image enhancement, and proper ROI size for MTF analysis. The target distance was 80 mm, which is the shortest distance at which the test chart provides adequate edge quality for reliable MTF measurement. MTF curves were calculated using the algorithm recommended by ISO 12233:2024 [8], and the equations summarized in Table 1 and Figure 2 were applied to convert spatial frequencies in different units across imaging spaces and domains.

### 4.1. Comparing MTF Curves at Two Different ROIs

**Figure 7:**
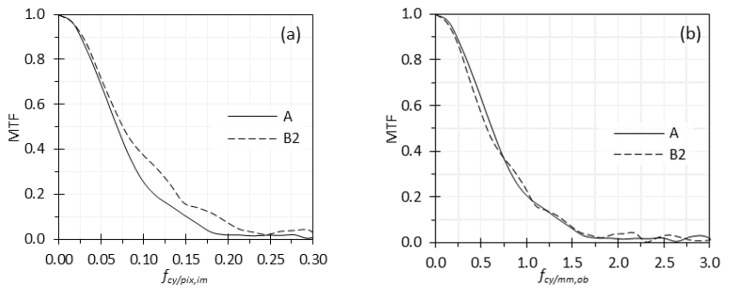
MTF curves at two different ROIs (A and B_2_ in Figure 4) and with spatial frequency expressed in two units: (**a**) cy/pix,im, and (**b**) cy/mm,ob.

In this case study, we compared the MTF curves obtained from two different ROIs in an endoscopic image, A and B_2_ (Figure 4). The MTF curves for A and B_2_ with different spatial-frequency units were compared in Figure 7. The default spatial-frequency unit used by the MTF calculation codes is cy/pix,im, and the results are shown in Figure 7a. It appeared that the MTF at B_2_ was higher than the MTF at A, with their MTF50 values (i.e., the spatial frequency at which the MTF drops to 50% of its zero-frequency value) of 0.079 and 0.069 cy/pix,im respectively. In other words, the edge MTF appeared higher than the center MTF, which is counterintuitive and inconsistent with visual observation. However, when the spatial-frequency unit was converted to cy/mm,ob, the trend reversed: the MTF at B_2_ became slightly worse than that at A, particularly at low spatial frequencies, with their MTF50 values of 0.58 and 0.62 cy/mm,ob, respectively. This outcome is more consistent with observation.

### 4.2. Comparing MTF Curves Based on Images with Different Dimensions

Image dimensions refer to the width and height of an image in pixels. Although “image size” is often used as an alternative term, it is not recommended because “image size” commonly refers to the disk space occupied by the image file rather than its pixel dimensions. Image dimensions influence sampling frequency and perceived sharpness, crucial for avoiding aliasing and ensuring accurate representation of high spatial frequencies. Output images of the same target from an endoscope can have different image dimensions due to varying scaling factors.

In this case study, we investigated the effect of image dimensions, and thus the scaling factor, on the MTF curves of our endoscopic system. We acquired images of the test chart using the endoscope at two different image dimensions—full (1280 × 1008 pixels) and medium (1090 × 858 pixels)—both capturing the same target area. Since the sensor pixel dimensions and in-camera resampling steps are considered proprietary by the company, we cannot independently verify the scaling factor. Therefore, we assume that the active sensor region and the full images contain the same number of pixels, i.e., a scaling factor of 1.

The analysis was conducted in two steps. First, we ignore the scaling factor difference between the full and medium images (i.e., assume they share the same scaling factor). Second, we applied the appropriate correction by noting that the medium image has a scaling factor equal to the full image’s scaling factor multiplied by 0.85, based on the ratio of their pixel dimensions. We calculated the MTF at the center for each set of images and plotted their MTF curves as a function of $f_{cy/mm,ob}$ in the same figure for comparison. $f_{cy/mm,ob}$ was calculated based on Equations (2), (3) and (7), assuming a pixel pitch of 2.8 microns and the measured absolute $M_{LT}$ described in Section 3.

**Figure 8:**
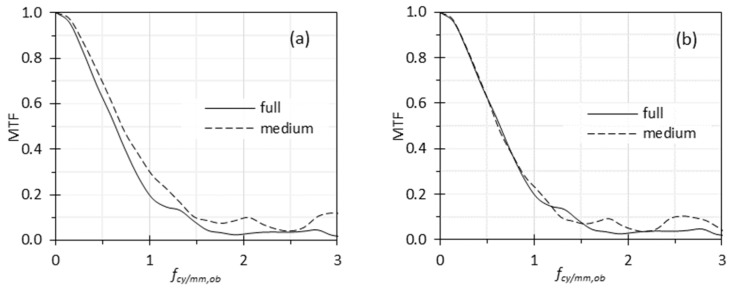
MTF curves derived from the full and medium images: (**a**) assuming the full and medium images have the same scaling factor; (**b**) applying a corrected scaling factor for the medium image equal to 85% of that of the full image.

When the full and medium images were assumed to share the same scaling factor of 1, the MTF calculated from the medium image appeared higher than that from the full image (Figure 8a), which is counterintuitive. Further analysis revealed that the same chart feature occupied different numbers of pixels in the two images. Since the magnification is determined by the optics and is fixed, this discrepancy indicates that the two images have different scaling factors. When an image is resampled through interpolation or other methods, the resulting image pixels no longer correspond directly to physical sensor pixels. Since Figure 8a was generated without accounting for the scaling difference between the full and medium images, the spatial-frequency values derived for the medium image are inaccurate.

When we recalculated the spatial-frequency values for the medium image using the correct scaling factor of 0.85, the corrected MTF for the medium image is shown in Figure 8b. This time, the full image and the medium image exhibited similar MTF curves, except that the curve for the medium image was noisier.

It should be noted that these results are based on the assumption that the full image has a scaling factor of 1. In practice, the full image may have been upscaled, meaning its scaling factor could be greater than 1. However, the ratio between the scaling factors of the full and medium images would remain unchanged, and, therefore, the relative positions of the two MTF curves would also remain the same, even though the absolute spatial-frequency values would differ. Thus, the same conclusion holds: a difference in scaling factor exists between the full and medium images and must be considered when comparing their MTF curves. Nevertheless, the absolute spatial frequency values should be interpreted with caution, given the underlying scaling assumption.

## 5. Discussions

The present findings build on our previous work on measuring spatially varying local magnification in endoscopic systems [2] and on methodologies for endoscope MTF measurement [16]. Those studies, however, did not examine how local magnification influences MTF interpretation, nor how the choice of spatial frequency units and image resampling affects MTF comparisons. By integrating MTF conversion equations with local magnification maps, the current study shows how incorrect assumptions about magnification, frequency units, or scaling factors can alter the apparent ranking of MTF curves across different ROIs or image formats. These results establish a systematic framework for converting MTF to object space units using appropriate local magnification values and sampling parameters.

In the first case study, the comparison of MTF curves from ROIs A and B_2_ (Figure 7) illustrates how different spatial frequency units can lead to conflicting interpretations. When spatial frequency is expressed in cy/pix,im unit, the MTF curve for ROI B_2_ appeared superior to that of ROI A. However, this trend reverses when spatial frequency is expressed in cy/mm,ob. This apparent contradiction arises because the cy/pix,im unit does not account for how geometric distortion alters effective sampling across the FOV. ROI at B_2_ has a smaller local tangential magnification than ROI at A, with $M_{LT}$($B_{2}$) = 0.83 $M_{LT}$(A), meaning that equal pixel spacings correspond to larger object space distances at B_2_ than at A. As a result, MTF expressed as a function of $f_{cy/pix,im}$ artificially inflates the apparent performance at the off-axis location B_2_. Converting the MTF to object space units while applying the correct position-dependent $M_{L}$ resolves this paradox and yields the expected relationship between ROIs A and B_2_. This discrepancy underscores that correct MTF interpretation across different image regions requires accurate spatial-frequency conversion parameters, including $M_{L}$. Although the result shown in Figure 7a could, in principle, arise from either a true optical effect or a unit-conversion artifact, the latter is the more likely explanation. In nearly all endoscopic images we have acquired, image quality at the center is visibly superior to that at the edge. Even if a true optical effect were present, the main conclusion remains unchanged: Spatial frequencies expressed in the digital image domain do not accurately represent object space sampling when $M_{L}$ varies across the FOV. Converting the MTF curves to object space units corrects this nonuniform sampling and enables a physically meaningful comparison of resolution performance across different ROIs.

The second case study (Figure 8) shows that differences in image dimensions—and the associated scaling factors—can lead to misleading MTF comparisons if not properly accounted for. The medium image initially appeared to exhibit better MTF; however, this result was an artifact caused by an incorrect scaling factor. Once the appropriate scaling factor was applied, the full and medium images yielded comparable MTF curves, with differences attributable primarily to noise. This finding highlights the importance of incorporating accurate scaling information when interpreting MTF results from resampled images. This case study should not be interpreted as an endorsement of MTF measurements based on downsampled images. Rather, it emphasizes the necessity of using correct scaling factors when comparing images with different dimensions. Downsampled images are typically generated from full-height images through processing steps such as interpolation, which can introduce artifacts and alter both spatial-frequency content and noise characteristics. Therefore, non-resampled full-height images are expected to provide the most reliable endoscope MTF measurements. It should be noted that this recommendation is based on comparisons between two processed output formats (“full” and “medium”) because raw, non-processed sensor data were not accessible for the commercial endoscope system used in this study. In practice, however, many commercial endoscopic systems apply in-camera processing—such as interpolation, sharpening, noise reduction, or scaling—which can influence both the measured MTF and the associated noise behavior. A rigorous validation of this recommendation would require direct comparison with MTF curves derived from raw sensor outputs, which would isolate the effects of in-camera processing and resampling. Therefore, this guidance should be interpreted as a practical recommendation: when raw images are unavailable, the least processed and highest-resolution image format provided by the system is expected to yield the most reliable MTF results. If only downsampled or highly processed images are available, the resulting MTF curves should be interpreted with caution, as such processing may distort apparent spatial-frequency content and obscure underlying system performance.

Pixel pitch and magnification are critical parameters that directly impact the spatial resolution of an imaging system. This study used a sensor pixel pitch of 2.8 microns from online sources, as most manufacturers consider this information proprietary. The true value may differ. Therefore, the resulting $M_{C}$ values and the focal-length estimate should be regarded as approximate checks of consistency rather than precise optical characterizations. If the pixel pitch of the image sensor is not available, the combined quantity of $M_{L}/P_{mm/pix,sen}$ can be measured using an accurate ruler or a grid target with a known grid size.

While this study focused on endoscopes, with all images captured using our endoscopic system, the equations and methods can be extended to a broad range of digital imaging systems. Endoscopes serve as a motivating example rather than a limitation of the method. Endoscopes differ from conventional digital cameras primarily in the severity of their geometric distortion, which leads to substantial variation in $M_{L}$ across the FOV [2]. As a result, the sampling density on the object plane varies with image radius, and the spatial-frequency conversion between the sensor plane and the object plane becomes position dependent. Accurate interpretation of MTF, therefore, requires a clear understanding of these optical characteristics. Similar challenges arise in fisheye and wide-angle cameras, as well as automotive surround-view systems, where strong geometric distortion causes spatially varying $M_{L}$ and sampling. For these systems, the procedure for obtaining $M_{L}$ and converting spatial frequencies across imaging spaces and the digital image domain—described in Section 3—applies directly. Even for cameras with minimal geometric distortion, the same conversion framework remains useful when expressing or comparing MTF in different spatial-frequency domains.

This study has several limitations. First, all data were obtained from a single endoscopic system at a single target distance, and the results may vary for systems with different optical designs or working distances. Second, no formal error-propagation analysis was performed; measurement variability in magnification, scaling, and chart alignment may introduce uncertainty in the converted spatial-frequency values. Finally, only two images are shown in each case study; they were selected as representative examples to illustrate how different spatial-frequency units and parameters can alter MTF interpretation. Despite these limitations, the underlying framework, equations, and methodology are general, and the qualitative conclusions remain valid. These limitations should be considered when generalizing the results or be further explored in future work.

## 6. Conclusions

Accurate conversion of image sampling and the corresponding spatial frequencies across different imaging spaces or domains is essential for evaluating an imaging system’s ability to resolve fine details and for comparing MTF across systems. In many endoscopic systems, captured images are resampled after initial detection by the sensor. As a result, sampling in the image sensor plane or digital image domain directly determines both the range and values of the spatial frequencies used for MTF analysis. For systems with significant geometric distortion, this work presents a practical framework to mitigate the impact of intra-image sampling variation on MTF interpretation by measuring local magnification and converting the spatial-frequency axis of the MTF into object space units at the corresponding ROIs.

This study underscores the importance of correctly converting and interpreting spatial frequencies across various imaging spaces or domains when analyzing endoscope MTF curves. The case studies demonstrate that improper spatial-frequency units can lead to misleading conclusions and highlight the need for accurate conversion parameters—such as pixel pitch, scaling factor, and local magnification—to ensure reliable results. By enabling accurate conversion of spatial frequencies among the object plane, sensor plane, and digital image domain—even in the presence of geometric distortion or resampling—the proposed framework supports meaningful integration of MTF results into longitudinal quality-assurance programs and device-to-device comparisons.

While clinical outcomes were not directly measured in this study, the methods developed here serve as essential steppingstones toward such investigations. For instance, in routine quality-assurance workflows, hospitals often compare imaging devices based on their ability to resolve clinically relevant features such as mucosal textures and vascular patterns, which correspond to specific object space spatial frequencies. Although endoscopes were used as the primary example because of their pronounced distortion and sampling variation, the spatial-frequency conversion framework developed here applies broadly to any imaging system in which magnification, distortion, or resampling affects MTF interpretation.

## Figures and Tables

**Table 1:** Summary of spatial frequencies, their conversion equations, and associated parameters.

Spaces	Terms	Units	Symbols or Equations
Digital image domain	Picture height	image pixels (pix,im)	$H_{pix,im}$
		mm on printed or displayed images (mm,im)	$H_{mm,im}$
	Spatial frequency	cycles per image pixel (cy/pix,im)	$f_{cy/pix,im}$
		cycle per picture height (cy/H)	$f_{cy/H}=f_{cy/pix,im}\cdot H_{pix,im}$
Image sensor plane	Pixel pitch	mm per sensor pixel (mm/pix,sen)	$P_{mm/pix,sen}$
	Picture height	sensor pixel (pix,sen)	$H_{pix,sen}$
		mm on the sensor (mm,sen)	$H_{mm,sen}$
	Spatial frequency	cycles per sensor pixel (cy/pix,sen)	$f_{cy/pix,sen}$
		cycles per mm on the sensor (cy/mm,sen)	$f_{cy/mm,sen}=f_{cy/pix,sen}/P_{mm/pix,sen}$
		cycle per picture height (cy/H)	$f_{cy/H}=f_{cy/pix,sen}\cdot H_{pix,sen}$ $f_{cy/H}=f_{cy/mm,sen}\cdot H_{mm,sen}$
Object plane	Spatial frequency	cycles per mm in the object plane (cy/mm,ob)	$f_{cy/mm,ob}$
	Angular spatial frequency	cycles per radian in object space (cy/rad,ob)	$f_{cy/rad,ob}=(z+\frac{r^{2}}{z})\cdot f_{cy/mm,ob}$ $f_{cy/rad,ob}\approx z\cdot f_{cy/mm,ob}$, if r≪z (i.e., at the FOV center).
		cycles per degree in object space (cy/deg,ob)	$f_{cy/deg,ob}=\frac{\pi }{180}\cdot f_{cy/rad,ob}$
Across imaging chain	Scaling factor (*s*) and related equations	dimensionless	$s=N_{pix,im}/N_{pix,sen}$
		cycles per sensor pixel (cy/pix,sen)	$f_{cy/pix,sen}=s\cdot f_{cy/pix,im}$
	Magnification (*M*) and related equations	dimensionless	M, might vary across different ROIs ($M_{L}$).
		cycles per mm on the object (cy/mm,ob)	$f_{cy/mm,ob}=f_{cy/mm,sen}\cdot M_{L}$ $f_{cy/mm,\;ob}=f_{\;cy/pix,sen}\cdot \frac{M_{L}}{P_{mm/pix,sen}}$
		sensor pixels per object mm (sen pix/ob mm)	$\frac{M_{L}}{P_{mm/pix,sen}}=\frac{N_{pix,sen}}{L}=\frac{N_{pix,im}}{s\cdot L}$ where *L* is the length of the target segment (in mm), and $N_{pix,sen}$ and $N_{pix,im}$ are the numbers of pixels corresponding to this length on the image sensor and in the digital image, respectively.

Note: Image/sensor/object pixel, mm, radian, and degree refer to quantities measured in digital image domain, sensor space, or object space, respectively.

## Data Availability

The original contributions presented in this study are included in the article. Further inquiries can be directed to the corresponding author.
